# Endovascular Treatment for a Huge Celiac Artery Aneurysm: 13-Year Follow-Up

**DOI:** 10.3400/avd.cr.26-00083

**Published:** 2026-08-20

**Authors:** Shigeo Ichihashi, Shinichi Iwakoshi, Katsuhiko Taira, Ayano Takeda, Toshihiro Tanaka

**Affiliations:** Department of Diagnostic and Interventional Radiology, Nara Medical University, Kashihara, Nara, Japan

**Keywords:** endovascular treatment, covered stent, visceral artery aneurysm

## Abstract

Celiac artery aneurysms are rare and carry a substantial risk of rupture. A 64-year-old woman was incidentally found to have a 38-mm celiac artery aneurysm on computed tomography. The left gastric and common hepatic arteries arising from the aneurysm were coil-embolized, and a 6 × 59-mm balloon-expandable covered stent was deployed from the celiac artery to the splenic artery. Postoperative imaging confirmed aneurysm exclusion and preserved splenic perfusion. At 13-year follow-up, computed tomography showed sustained stent patency and aneurysm shrinkage from 38 to 31 mm without complications, supporting the long-term durability of endovascular treatment in anatomically suitable patients.

## Introduction

Celiac artery aneurysms (CAAs) are rare, comprising approximately 4%–5% of visceral artery aneurysms (VAAs).^1,2)^ Despite being often asymptomatic, untreated CAAs carry a high rupture risk. Rupture has been reported to occur in approximately 5% of celiac trunk aneurysms ranging from 15 to 22 mm in diameter and in 50% to 70% of those that exceed 32 mm in diameter.^3,4)^ Although open surgical repair has been the traditional standard, endovascular treatment (EVT) has become increasingly preferred due to lower morbidity and comparable mid-term outcomes. In a recent guideline, endovascular rather than open repair is recommended for patients with a VAA who are anatomically suitable.^5)^ However, long-term durability data of EVT, particularly beyond 10 years, are limited. We report a case of CAA successfully treated with EVT using a covered stent and coil embolization, demonstrating excellent outcomes over 13 years.

## Case Report

**Fig. 1:**
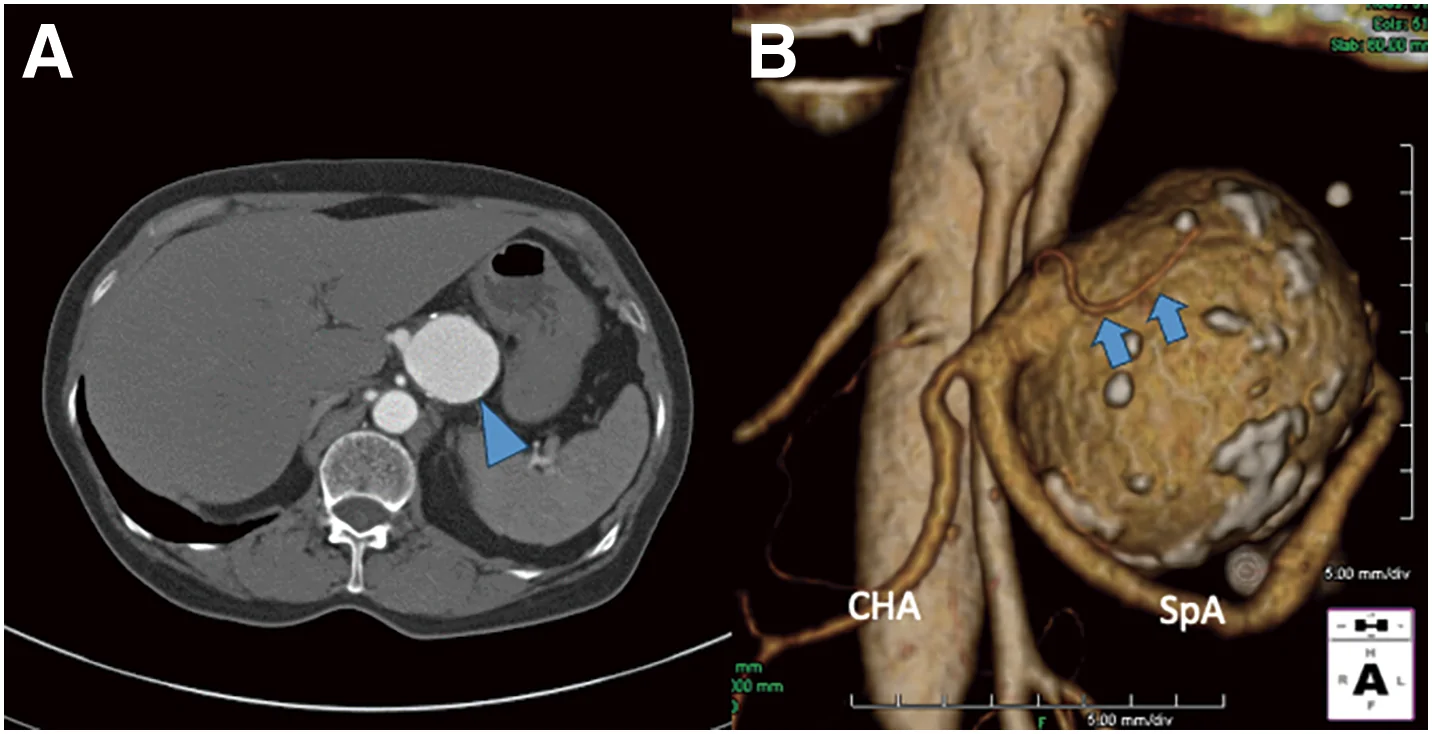
Preoperative CT. CT shows a huge celiac artery aneurysm (arrowhead). Distal to the aneurysm, the splenic artery and common hepatic artery bifurcated, whereas the left gastric artery arose from the aneurysm sac (arrow). (**A**) Axial image. (**B**) Volume rendering image. CHA, common hepatic artery; SpA, splenic artery; CT, computed tomography

**Fig. 2:**
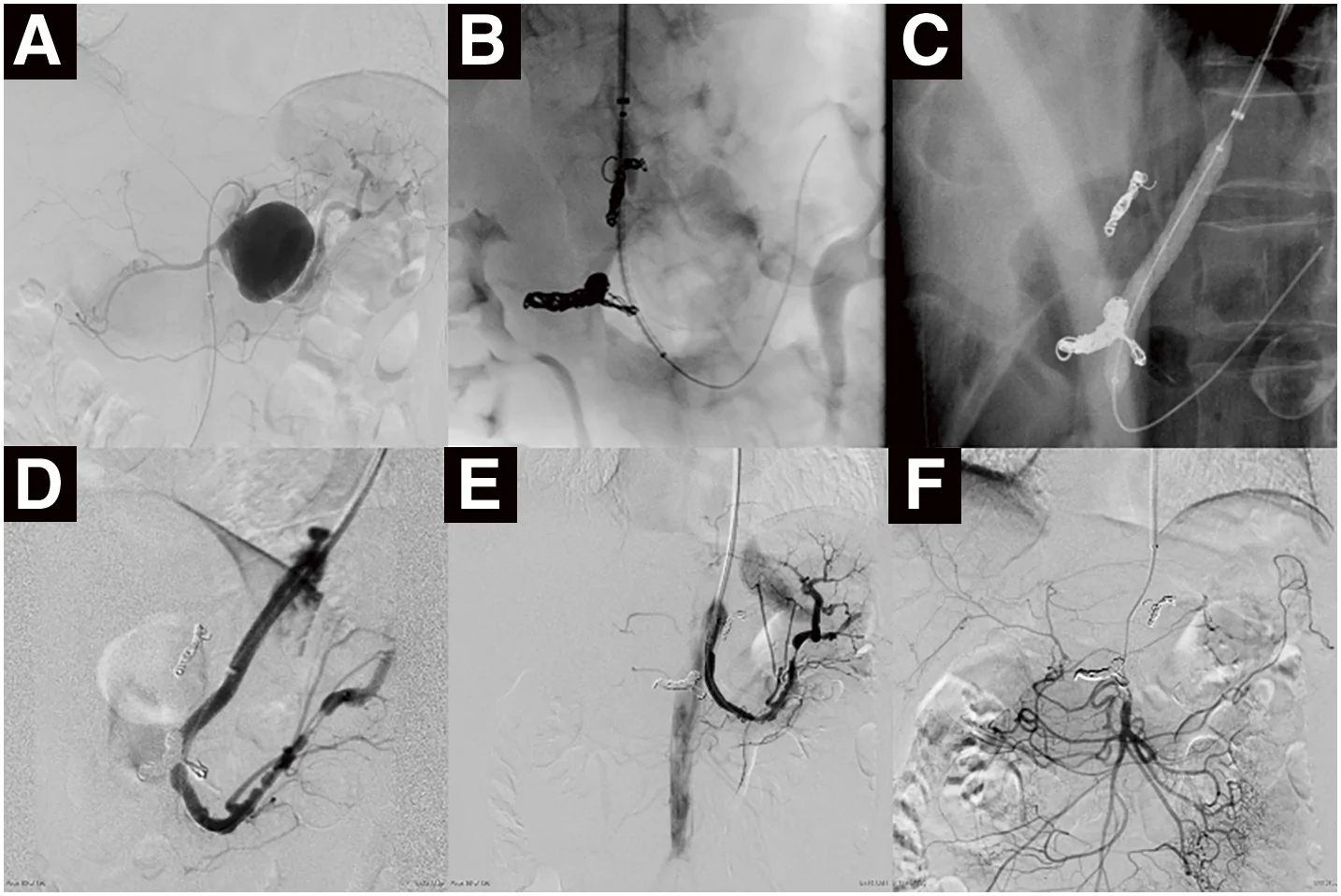
Endovascular procedure. (**A**) Celiac trunk angiography showed a large saccular aneurysm arising from the celiac trunk. (**B**) After embolization of the CHA and LGA, an Advanta V12 covered stent was positioned across the aneurysm from the celiac trunk to the SpA. (**C**) The covered stent was then deployed. (**D**) After covered stent placement, splenic artery flow was preserved. (**E**) Celiac trunk angiography demonstrated complete exclusion of the aneurysm. (**F**) SMA angiography demonstrated preserved hepatic arterial flow through the pancreatic arcade. CHA, common hepatic artery; LGA, left gastric artery; SMA, superior mesenteric artery; SpA, splenic artery

**Fig. 3:**
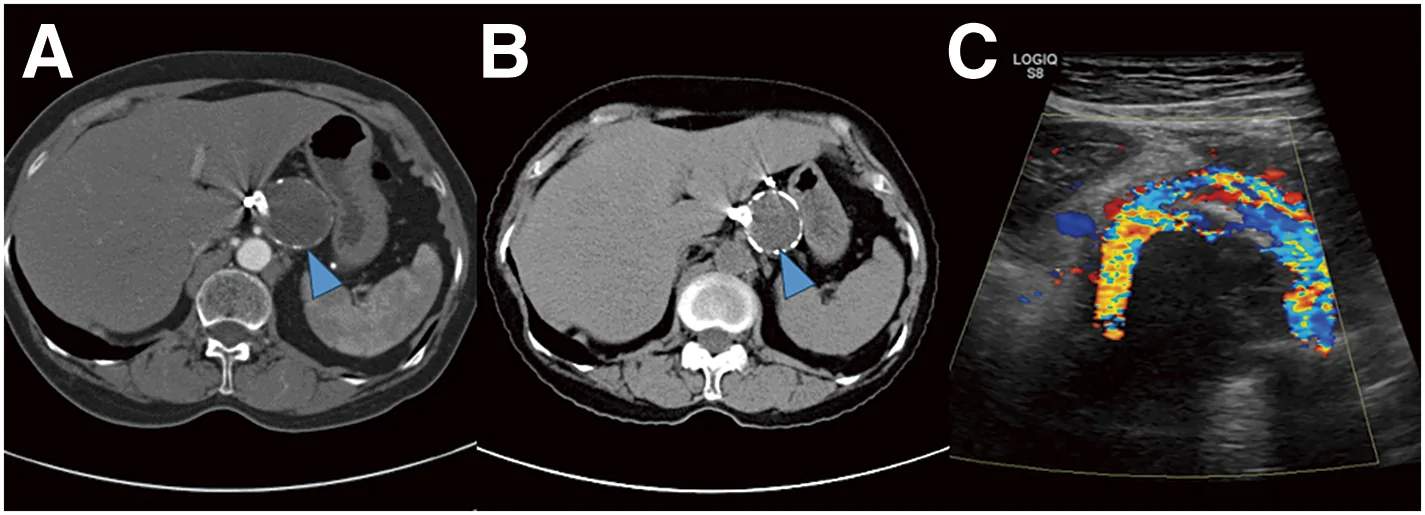
Follow-up images. (**A**) Postoperative CT showed no endoleak within the aneurysm (arrowhead). (**B**) CT obtained 13 years after EVT showed aneurysm shrinkage (arrowhead). (**C**) DUS obtained 13 years after EVT demonstrated patency of the covered stent. DUS, duplex ultrasound; EVT, endovascular treatment

A 64-year-old woman underwent contrast-enhanced computed tomography (CT) for bladder stone evaluation, revealing a 38-mm celiac artery aneurysm. The left gastric artery (LGA) originated from the aneurysmal segment, with distal bifurcation to the splenic artery (**Fig. 1**). In view of the aneurysm diameter and the associated rupture risk, EVT consisting of covered stent placement with branch embolization was performed. The right common femoral artery (CFA) was punctured, and a 6-F Ansel-type II sheath (Cook Medical, Bloomington, IN, USA) was inserted. A 4-F-long sheath was placed via the left side. Angiography of the celiac artery revealed an aneurysm (**Fig. 2**). It was difficult to cannulate the celiac artery with the Ansel-type II sheath because the celiac origin was severely downward-angulated. Therefore, brachial access was considered more favorable. A 4.5-F Parent sheath was advanced via the left brachial artery, which allowed stable catheterization of the celiac artery. Through the right CFA sheath, a 2.8-F Attendant occlusion balloon catheter (Terumo, Tokyo, Japan) was positioned at the celiac origin for flow control. A microcatheter was advanced via the left brachial access into the common hepatic artery (CHA), and coil embolization of the CHA and dorsal pancreatic artery (DPA) was performed using IDC coils (two 10 mm × 20 cm coils, three 8 mm × 20 cm coils; Boston Scientific, Marlborough, MA, USA) and an Interlock coil (one 8 mm × 20 cm coil; Boston Scientific). Selection of the LGA was possible with an RLG catheter. A 2.2-F Estream microcatheter (Toray Medical, Tokyo, Japan) was advanced into the LGA and was embolized with two 4-mm × 12 cm IDC coils and 4 Hiral coils (Cook Medical). The sheath was upsized to a 7-F Shuttle (Cook Medical), which was advanced into the splenic artery. A 6 × 59-mm covered stent (Advanta V12; Getinge, Gothenburg, Sweden) was deployed from the distal celiac artery to the splenic artery, with the proximal edge of the stent positioned just distal to the celiac ostium. Completion angiography demonstrated preserved splenic artery flow and complete exclusion of the aneurysm. Superior mesenteric artery (SMA) angiography confirmed preserved gastric and hepatic perfusion through collateral pathways. The procedure was completed without immediate complications. After the procedure, dual anti-platelet therapy with clopidogrel and aspirin was administered for 6 months, followed by lifelong aspirin (300 mg per day) to prevent stent-graft thrombosis. Follow-up CT after EVT demonstrated the preserved arterial flow into the splenic artery via the covered stent and no endoleak inside the aneurysm (**Fig. 3**). The diameter of the aneurysm shrunk from 38 mm postoperatively to 33 mm at 5 years and 31 mm at 7 years, and remained the same at 13 years of follow-up. Arterial flow through the covered stent remained preserved at 13 years of follow-up.

## Discussion

The present case highlights the long-term durability of endovascular repair for a celiac artery aneurysm using a covered stent. Several endovascular strategies have been described for the management of CAAs, including direct coil packing of the aneurysm sac, isolation of the parent artery, and preservation of the parent vessel with a covered stent.^6,7)^ Procedural strategy needs to be tailored to aneurysm location, morphology, collateral circulation, and vessel characteristics. Sac-packing embolization was mainly used for VAAs with a narrow neck, the isolation technique for more complex lesions involving bifurcations or tortuous segments, and covered stents or stent-assisted coiling for major trunks where distal perfusion had to be preserved. In this patient, the splenic artery demonstrated a caliber comparable to that of the celiac trunk, providing an optimal proximal/distal landing zone and facilitating the covered stent method. A covered stent was deployed from the celiac trunk to the splenic artery to maintain arterial perfusion while excluding the aneurysm. To prevent a potential type II endoleak, both the LGA and the CHA were selectively coil-embolized prior to stent deployment. The treatment successfully excluded the aneurysm while maintaining arterial flow. Although no universally standardized antithrombotic regimen has been established for EVT of abdominal VAAs, the current CIRSE Standards of Practice recommend dual antiplatelet therapy after covered stent placement, followed by long-term aspirin monotherapy. In this case, dual antiplatelet therapy with clopidogrel and aspirin was administered for 6 months, followed by lifelong aspirin monotherapy, which is consistent with the later CIRSE Standards of Practice.

Venturini et al. compared technical and clinical success rates between covered stenting and embolization and they concluded that both have similar technical success rates (97% vs. 96%, respectively), with a slightly better clinical success rate in favor of covered stents (87% vs. 81.4%).^8)^ The potential drawbacks of covered stents include the size and rigidity of delivery systems that preclude deployment in a tortuous and small diameter vessel, with reported stent patency of 88% at a median follow-up of 32.8 months. In another study, a series of 40 VAAs treated with Viabahn stent grafts (W. L. Gore & Associates, Flagstaff, AZ, USA), including 6 cases involving the celiac trunk, were evaluated.^9)^ Among the 29 patients with available imaging follow-up, the mean follow-up duration was 20.7 months. Two patients developed early stent-graft thrombosis at 1 and 3 months after treatment. At 3 years, imaging follow-up was available for 7 patients, with all maintaining complete aneurysm exclusion and stent-graft patency. Qiu et al. also reported the results of a multicenter study of 65 patients who underwent covered stent placement for VAAs.^10)^ After a mean follow-up of 20 months, mild restenosis was observed in 4 stents (7%), whereas patency was preserved in the remaining covered stents. While these results support the feasibility and effectiveness of covered stent repair for VAAs, the available follow-up in the literature remains relatively short. The present case, with durable stent patency and aneurysm regression over 13 years, provides valuable evidence reinforcing endovascular therapy as a durable long-term treatment option for CAAs.

This report has several limitations. First, it describes a single case, and therefore the findings cannot be generalized to all patients with CAAs. Further accumulation of cases with long-term follow-up is necessary to better define the durability and safety of this strategy. Second, covered stent placement is highly dependent on vascular anatomy, including adequate proximal and distal landing zones, appropriate vessel diameter, and limited tortuosity. Therefore, this approach may be feasible only in selected patients with favorable anatomy.

## Conclusion

This case suggests that EVT with covered stent-graft exclusion and adjunctive coil embolization may be a durable treatment option for CAAs in anatomically suitable patients.
